# Reproductive Factors and Endometrial Cancer Risk Among Women

**DOI:** 10.1001/jamanetworkopen.2023.32296

**Published:** 2023-09-05

**Authors:** Ryoko Katagiri, Motoki Iwasaki, Sarah Krull Abe, Md. Rashedul Islam, Md. Shafiur Rahman, Eiko Saito, Melissa A. Merritt, Ji-Yeob Choi, Aesun Shin, Norie Sawada, Akiko Tamakoshi, Woon-Puay Koh, Ritsu Sakata, Ichiro Tsuji, Jeongseon Kim, Chisato Nagata, Sue K. Park, Sun-Seog Kweon, Xiao-Ou Shu, Yu-Tang Gao, Shoichiro Tsugane, Takashi Kimura, Jian-Min Yuan, Seiki Kanemura, Yukai Lu, Yumi Sugawara, Keiko Wada, Min-Ho Shin, Habibul Ahsan, Paolo Boffetta, Kee Seng Chia, Keitaro Matsuo, You-Lin Qiao, Nathaniel Rothman, Wei Zheng, Manami Inoue, Daehee Kang

**Affiliations:** 1 Division of Cohort Research, National Cancer Center Institute for Cancer Control, Tokyo, Japan; 2 National Institute of Health and Nutrition, National Institutes of Biomedical Innovation, Health and Nutrition, Tokyo, Japan; 3 Division of Epidemiology, National Cancer Center Institute for Cancer Control, Tokyo, Japan; 4 Division of Prevention, National Cancer Center Institute for Cancer Control, Tokyo, Japan; 5 Hitotsubashi Institute for Advanced Study, Hitotsubashi University, Tokyo, Japan; 6 Research Center for Child Mental Development, Hamamatsu University School of Medicine, Shizuoka, Japan; 7 Institute for Global Health Policy Research, National Center for Global Health and Medicine, Tokyo, Japan; 8 The Daffodil Centre, The University of Sydney, a joint venture with Cancer Council NSW, Sydney, New South Wales, Australia; 9 Department of Biomedical Sciences, Seoul National University Graduate School, Seoul, Korea; 10 Cancer Research Institute, Seoul National University, Seoul, Korea; 11 Department of Preventive Medicine, Seoul National University College of Medicine, Seoul, Korea; 12 Department of Public Health, Hokkaido University Faculty of Medicine, Sapporo, Japan; 13 Healthy Longevity Translational Research Programme, Yong Loo Lin School of Medicine, National University of Singapore, Singapore; 14 Singapore Institute for Clinical Sciences, Agency for Science Technology and Research, Singapore; 15 Radiation Effects Research Foundation, Hiroshima, Japan; 16 Tohoku University Graduate School of Medicine, Miyagi, Japan; 17 Graduate School of Cancer Science and Policy, National Cancer Center, Goyang, Korea; 18 Department of Epidemiology and Preventive Medicine, Gifu University Graduate School of Medicine, Gifu, Japan; 19 Department of Preventive Medicine, Chonnam National University Medical School, Gwangju, Korea; 20 Division of Epidemiology, Vanderbilt-Ingram Cancer Center, Vanderbilt Epidemiology Center, Vanderbilt University Medical Center, Nashville, Tennessee; 21 Department of Epidemiology, Shanghai Cancer Institute, Shanghai, China; 22 Division of Cancer Control and Population Sciences, UPMC Hillman Cancer Center, University of Pittsburgh, Pittsburgh, Pennsylvania; 23 Department of Epidemiology, Graduate School of Public Health, University of Pittsburgh, Pittsburgh, Pennsylvania; 24 Department of Public Health Sciences, University of Chicago, Chicago, Illinois; 25 Stony Brook Cancer Center, Stony Brook University, Stony Brook, New York; 26 Department of Medical and Surgical Sciences, University of Bologna, Bologna, Italy; 27 Saw Swee Hock School of Public Health, National University of Singapore, Singapore; 28 Division Cancer Epidemiology and Prevention, Aichi Cancer Center Research Institute, Nagoya, Japan; 29 Department of Cancer Epidemiology, Nagoya University Graduate School of Medicine, Nagoya, Japan; 30 School of Population Medicine and Public Health, Chinese Academy of Medical Sciences and Peking Union Medical College, Beijing, China; 31 Division of Cancer Epidemiology and Genetics, Occupational and Environmental Epidemiology Branch, National Cancer Institute, Bethesda, Maryland; 32 Seoul National University College of Medicine, Seoul, Korea

## Abstract

**Importance:**

Despite evidence of an association between reproductive factors and endometrial cancer risk, prospective studies have been conducted mainly in non-Asian countries.

**Objective:**

To assess the association between reproductive factors, such as number of deliveries, age at menarche, or menopause, and endometrial cancer risk.

**Design, Setting, and Participants:**

This cohort study used pooled individual data from 13 prospective cohort studies conducted between 1963 and 2014 in the Asia Cohort Consortium. Participants were Asian women. Data analysis was conducted from September 2019 to April 2023.

**Exposures:**

Reproductive factors were assessed using a questionnaire in each cohort.

**Main Outcomes and Measures:**

The main outcome was time to incidence of endometrial cancer. A Cox proportional hazards model was used to calculate hazard ratios (HRs) and 95% CIs.

**Results:**

A total of 1005 endometrial cancer cases were detected among 332 625 women (mean [SD] age, 54.3 [10.4] years) during a mean (SD) of 16.5 (6.4) years of follow-up. Increasing number of deliveries was associated with a decreased endometrial cancer risk in a dose-response manner (≥5 deliveries vs nulliparous [reference]: HR, 0.37; 95% CI, 0.26-0.53; *P *for trend < .001). Compared with menarche at younger than 13 years, menarche at 17 years or older had an HR of 0.64 (95% CI, 0.48-0.86; *P *for trend < .001). Late menopause (age ≥55 years) showed an HR of 2.84 (95% CI, 1.78-4.55; *P *for trend < .001) compared with the youngest age category for menopause (<45 years). Age at first delivery, hormone therapy, and breastfeeding were not associated with endometrial cancer risk.

**Conclusions and Relevance:**

This large pooled study of individual participant data found that late menarche, early menopause, and a higher number of deliveries were significantly associated with a lower risk of endometrial cancer. These convincing results from Asian prospective studies add to the growing body of evidence for the association between reproductive factors and endometrial cancer.

## Key Points

**Question:**

Are reproductive factors and endometrial cancer risk associated among individuals in East Asia?

**Findings:**

In this pooled cohort study of 332 625 women including 1005 endometrial cancer cases from 13 Asian cohort studies, late menarche, early menopause, and a greater number of deliveries were associated with a lower risk of endometrial cancer.

**Meaning:**

The findings from this large pooled analysis in Asia, which are consistent with previous evidence, may have an impact on the understanding of risk factors for endometrial cancer.

## Teaser

This cohort study assesses the association of reproductive factors, such as number of deliveries, age at menarche, and age at menopause, with endometrial cancer risk among East Asian women.

## Introduction

Endometrial cancer is the sixth most common cancer among women worldwide and accounted for 2% of new cancer cases and 1% of deaths in 2020.^1^ The region-specific incidence varies worldwide; the age-standardized rate (per 100 000) was 21.1 in North America and 8.2 in Eastern Asia.^2,3^ In addition to regional variations, the incidence rate differs according to race. According to the Surveillance, Epidemiology, and End Results Program in the United States, the incidence rate was 28.0 among non-Hispanic White people and 22.6 among Asian and Pacific Islander people.^4^ Although previous studies have prospectively investigated reproductive factors and the risk of endometrial cancer in the United States and Europe,^5,6,7^ limited studies in this regard have emanated from Asian countries.^8^

The endometrium modifies its structure in response to the menstrual cycle, with fluctuations in estrogen and progesterone levels. It has been hypothesized that unopposed estrogen exposure leads to endometrial hyperplasia, which increases the risk of endometrial cancer.^9,10^ Reproductive factors, such as parity, age at menarche, and menopause, are potentially associated with differences in unopposed estrogen exposure among individuals and might lead to different endometrial cancer risks. Prospective studies have examined these associations,^5,6,7^ and meta-analyses including case-control and prospective studies have been published.^11,12,13,14,15^ According to an umbrella review, which included 171 meta-analyses of 1354 individual studies of 53 risk factors, parity, higher age at last birth, and higher age at menarche were associated with a decreased risk of endometrial cancer.^16^ Moreover, many previous studies have shown that multiparity, higher parity, and early menopause are associated with a decreased risk of endometrial cancer.^14,15^ However, the association of some factors, such as age at the first pregnancy or breastfeeding, with endometrial cancer risk has been controversial in prospective studies.^5,6,7,14^

In Asian countries, investigating factors associated with endometrial cancer in a single prospective cohort is likely to be difficult because the incidence rate of endometrial cancer is relatively low.^2,3^ Therefore, in this study, we pooled 13 Asian prospective cohort studies in the Asia Cohort Consortium (ACC), aiming to investigate the association between reproductive factors and endometrial cancer with a large sample size.

## Methods

### Study Population

ACC is a consortium of prospective studies conducted in Asia. The details of the ACC have been described elsewhere.^17,18,19^ Thirteen cohort studies were included in the endometrial cancer project, among which 8 were conducted in Japan,^20,21,22,23,24,25,26,27^ 3 were conducted in Korea,^28,29,30^ 1 in China,^31^ and 1 in Singapore.^32^ These cohort studies provided information on reproductive factors, the incidence of endometrial cancer, follow-up duration, and potential confounders, such as age, current smoking status, and alcohol drinking status at the baseline. We excluded participants with the following characteristics: male sex (n = 241 676); missing data regarding sex and age (n = 2422); history of endometrial cancer at the baseline survey (n = 1039); follow-up duration of 0 or less or missing (n = 1858); history of hysterectomy at baseline (n = 10 004); body mass index (BMI [calculated as weight in kilograms divided by height in meters squared]) of less than 14 or greater than 45 (n = 7571); and missing data on parity (n = 15 884). A total of 332 625 participants were included in this study.

### Ethics Approval and Consent to Participate

The institutional review board of the National Cancer Center Japan approved the protocol for the analysis in the ACC. Each cohort study protocol, including that pertaining to informed consent, was approved by the respective institutional ethics committees. Reporting of this study followed the Strengthening the Reporting of Observational Studies in Epidemiology (STROBE) reporting guideline.

### Exposure, Outcome, and Other Variables

Our working group in the ACC cleaned the data to harmonize responses to different questions in the participating cohort studies. The detailed process is described in the eAppendix in Supplement 1. For example, given that the response to number of deliveries was categorical in 2 participating studies, we used the categorical values in the analysis and defined the cutoff values included in the original categories. Regarding the question about pregnancy, we focused on the number of deliveries (full-term births) rather than the number of pregnancies because most cohorts asked this. Moreover, we defined the plausible range of the age at first delivery (10-49 years), age at menarche (10-23 years), and menopause (≥20 years), and responses to age beyond this range were treated as missing so as not to include implausible answers in the analyses.

Regarding reproductive factors, questions pertaining to pregnancy (never and ever), the number of deliveries (0, 1-2, 3-4, and ≥5 times), age at first delivery (≤20, 21-25, 26-30, and ≥31 years or nulliparous), age at menarche (<13, 13-14, 15-16, and ≥17 years), menopausal status (premenopausal and postmenopausal), age at menopause (<45, 45-49, 50-54, and ≥55 years), breastfeeding status (never and ever), and hormone therapy (HT) status (never and ever) were asked in the baseline questionnaire of each prospective study. A limited number of cohorts included questions pertaining to breastfeeding and HT status. Since oral contraceptive (OC) use (never or ever) was asked in 5 cohorts, we did not assess the association of OC use with the risk of endometrial cancer. Also, since the number of deliveries was not assessed in the Life Span Study (LSS), results regarding the number of deliveries were not included. In addition, the Japan Collaborative Cohort Study (JACC) and LSS included questions about age at first pregnancy (not delivery).

The incidence of endometrial cancer was determined in each prospective study via linkage to local cancer registries. The *International Classification of Diseases for Oncology, Third Edition*, codes for endometrial cancer are C54 and C55.9. First primary cancer was included in this study. Information on whether the endometrial cancer was primary, secondary, or more was collected in each cohort and submitted to the study coordinating center. Because information regarding morphological and histological codes was submitted for a limited number of cohorts, topological codes were used for the definition. Age (continuous), BMI (continuous), current smoking status (yes and no), and current alcohol consumption status at the baseline (yes and no) were considered as possible confounding variables in the model.

### Statistical Analysis

This analysis was conducted from September 2019 to April 2023. In the present analysis, we examined the association between reproductive factors and endometrial cancer incidence using hazard ratios (HRs) and 95% CIs in the Cox proportional hazards model. The shared frailty model was applied to pooled individual data, and we used the variable of a cohort in the RANDOM statement of the PHREG procedure in SAS software.^33^ We created 2 statistical models: age-adjusted and multivariable-adjusted. The age-adjusted model incorporated adjustment for age at the baseline, while the multivariable-adjusted model incorporated adjustment for age at the baseline, current smoking status, current drinking status, and other reproductive factors (age at menarche and menopause were adjusted in the model of parous status, number of deliveries, and age at first delivery; age at menopause and parous status were adjusted in the model of age at menarche; age at menarche and parous status were adjusted in the model for age at menopause; parous status, age at menarche, and menopause were adjusted in the model for HT use and breastfeeding). Regarding missing values for menopausal status, we defined postmenopausal when participants’ menopausal status was missing and their age was 54 years or older and those aged 44 years or younger were defined as premenopausal because the median age at menopause is approximately 50 years among Japanese women, and these statuses were plausible.^34^ On the other hand, the menopausal status of participants with age 45 to 53 years was left as a missing at the time of this variable cleaning. Next, 5 rounds of multiple imputation were performed on the pooled individual data for missing adjusted variables. Reproductive factors, age at the baseline, BMI, follow-up duration, and diagnosis of endometrial cancer were included in the multiple imputation procedure in SAS, and the Markov chain Monte Carlo method was used. The 5 calculated estimates were combined into the final estimates according to Rubin rule using the SAS MIANALYZE procedure. To calculate the *P* for trend, we used the mean value of each category when there was a range of values in that category. For example, for age 45 to 49 years in the second category of age at menopause, we applied 47 to calculate *P *for trend. We performed stratified analyses classified by BMI (greater or less than the median), menopausal status, and parity. In addition, because nulliparous women could not be distinguished from women who did not report age at first pregnancy in LSS, we further examined associations excluding LSS to confirm the consistency of the results. All statistical analyses were performed using SAS version 9.4 (SAS Institute Inc), and *P* < .05 indicated statistical significance.

## Results

Among 332 625 women (mean [SD] age, 54.3 [10.4] years at baseline) from 13 cohort studies, a total of 1005 endometrial cancer cases were observed. The baseline characteristics of the cohort studies are presented in Table 1. The mean participants’ age at baseline ranged from 49.5 to 61.2 years. More than 90% of the participants experienced pregnancy, except those included in the LSS. A majority of participants reported experiencing menarche at age 13 to 16 years and menopause at age 45 to 54 years.

**Table 1.** Characteristics of Participants in the Included Cohort Studies

Characteristic	Participants, No. (%)													
	SWHS	JPHC1	JPHC2	JACC	Miyagi	Ohsaki	LSS	Takayama	3P Miyagi	KMCC	KNCC	Namwon	SCHS	Total
No. of women after exclusion	74 892	21 073	27 094	40 836	21 903	21 043	29 886	14 491	15 635	8640	15 586	6277	35 269	332 625
Endometrial cancer cases, No.	303	97	67	63	89	47	105	39	25	10	25	6	129	1005
Baseline year	1997-2000	1990-1992	1993-1995	1988-1990	1990	1995	1963-1993	1992	1984	1994-2004	2002-2014	2004-2007	1993-1999	NA
Country	China	Japan	Japan	Japan	Japan	Japan	Japan	Japan	Japan	Korea	Korea	Korea	Singapore	NA
Duration of follow-up, mean (SD), y	17.3 (3.1)	21.5 (3.7)	18.4 (3.5)	16.4 (5.5)	22.2 (5.5)	11.0 (4.2)	23.4 (10.3)	14.0 (3.8)	7.8 (2.5)	14.3 (4.2)	9.1 (3.3)	12.7 (2.1)	14.3 (3.5)	16.5 (6.4)
Age, mean (SD), y	52.6 (9.1)	49.6 (5.9)	54.2 (8.8)	57.3 (10.0)	52.0 (7.4)	60.2 (10.0)	52.0 (15.1)	55.4 (13.2)	56.9 (11.2)	54.3 (13.8)	49.5 (9.1)	61.2 (7.9)	56.3 (8.0)	54.3 (10.4)
BMI, mean (SD)	24.0 (3.4)	23.6 (3.1)	23.4 (3.2)	22.9 (3.1)	23.7 (3.1)	23.7 (3.2)	22.0 (3.5)	22.0 (2.9)	23.3 (3.4)	24.0 (3.4)	23.0 (3.0)	24.6 (3.2)	23.2 (3.3)	23.3 (3.3)
Current smoker	1783 (2.4)	1201 (5.7)	1719 (6.3)	1797 (4.4)	1459 (6.7)	1372 (6.5)	3896 (13.0)	1673 (11.5)	1018 (6.5)	590 (6.8)	674 (4.3)	288 (4.6)	2198 (6.2)	19 668 (5.9)
Current alcohol drinker	1453 (1.9)	4860 (23.1)	5405 (19.9)	8851 (21.7)	4647 (21.2)	4086 (19.4)	7798 (26.1)	9564 (66.0)	3727 (23.8)	1504 (17.4)	6543 (42.0)	2151 (34.3)	3166 (9.0)	63 755 (19.2)
Family history of endometrial cancer	NA	301 (1.4)	381 (1.4)	197 (0.5)	488 (2.2)	NA	NA	NA	318 (2.0)	NA	72 (0.5)	NA	NA	1757 (0.5)
Ever pregnant	72 392 (96.7)	19 914 (94.5)	25 523 (94.2)	39 314 (96.3)	21 360 (97.5)	20 321 (96.6)	21 149 (70.8)	13 254 (91.5)	14 129 (90.4)	8180 (94.7)	15 046 (96.5)	6242 (99.4)	32 766 (92.9)	309 590 (93.1)
Ever received HT	2650 (3.5)	NA	NA	NA	1412 (6.4)	1578 (7.5)	NA	219 (1.5)	NA	270 (1.4)	2145 (13.8)	672 (10.7)	1300 (3.7)	10 246 (3.1)
Ever breastfed	NA	16 770 (79.6)	22 017 (81.3)	NA	16 861 (77.0)	17 033 (81.0)	NA	NA	NA	7136 (82.6)	11 557 (74.1)	6046 (96.3)	NA	97 420 (29.3)
No. of deliveries														
0	2500 (3.3)	1159 (5.5)	1570 (5.8)	1522 (3.7)	543 (2.5)	722 (3.4)	NA	1237 (8.5)	1506 (9.6)	460 (5.3)	541 (3.5)	35 (0.6)	2503 (7.1)	14 298 (4.3)
1-2	56 675 (75.7)	8794 (41.7)	10 899 (40.2)	17 849 (43.7)	10 690 (48.8)	8801 (41.8)	NA	8252 (56.9)	6233 (39.9)	1790 (20.7)	11 308 (72.5)	840 (13.4)	9925 (28.1)	152 056 (45.7)
3-4	12 504 (16.7)	9092 (43.1)	10 646 (39.3)	17 996 (44.1)	9841 (44.9)	9477 (45.0)	NA	4232 (29.2)	5379 (34.4)	3265 (37.8)	3449 (22.1)	2682 (42.7)	13043 (37.0)	101 606 (30.5)
≥5	3213 (4.3)	1837 (8.7)	3661 (13.5)	3469 (8.5)	829 (3.8)	2043 (9.7)	NA	770 (5.4)	2517 (16.1)	3031 (35.1)	289 (1.9)	2709 (43.2)	9798 (27.8)	34 166 (10.3)
Age at first delivery, y														
≤20	8520 (11.4)	1630 (7.7)	1832 (6.8)	1901 (4.7)	1554 (7.1)	1739 (8.3)	4738 (15.9)	658 (4.5)	1406 (9.0)	1664 (19.3)	330 (2.1)	1600 (25.5)	6846 (19.4)	34 418 (10.3)
21-25	22 892 (30.6)	10 574 (50.2)	14 271 (52.7)	21 193 (51.9)	14 221 (64.9)	13 863 (65.9)	11 710 (39.2)	7376 (50.9)	8520 (54.5)	4733 (54.8)	5341 (34.3)	3800 (60.5)	13 522 (38.3)	152 016 (45.7)
26-30	33 168 (44.3)	6013 (28.5)	7079 (26.1)	11 903 (29.1)	4711 (21.5)	3497 (16.6)	3708 (12.4)	4034 (27.8)	3139 (20.1)	1268 (14.7)	7552 (48.4)	684 (10.9)	8878 (25.2)	95 634 (28.8)
≥31	7807 (10.4)	1253 (5.9)	1337 (4.9)	1787 (4.4)	746 (3.4)	570 (2.7)	993 (3.3)	786 (5.4)	734 (4.7)	211 (2.4)	1345 (8.6)	104 (1.7)	3496 (9.9)	21 169 (6.4)
Nulliparous or missing	2505 (3.3)	1603 (7.6)	2575 (9.5)	4052 (9.9)	671 (3.1)	1374 (6.5)	8737 (29.2)	1636 (11.3)	1836 (11.7)	764 (8.8)	1019 (6.5)	89 (1.4)	2527 (7.2)	29 388 (8.8)
Age at menarche, y														
<13	4691 (6.3)	1873 (8.9)	2445 (9.0)	2537 (6.2)	1733 (7.9)	472 (2.2)	1321 (4.4)	1862 (12.9)	881 (5.6)	102 (1.2)	544 (3.5)	247 (3.9)	5043 (14.3)	23 751 (7.1)
13-14	27 327 (36.5)	8610 (40.9)	10 620 (39.2)	14 264 (34.9)	8260 (37.7)	3222 (15.3)	8603 (28.8)	5287 (36.5)	5175 (33.1)	1031 (11.9)	2713 (17.4)	1293 (20.6)	13 545 (38.4)	109 950 (33.1)
15-16	29 532 (39.4)	7378 (35.0)	8845 (32.6)	14 867 (36.4)	6883 (31.4)	4825 (22.9)	8193 (27.4)	4952 (34.2)	6036 (38.6)	2824 (32.7)	2644 (17.0)	1417 (22.6)	12 164 (34.5)	11 0560 (33.2)
≥17	13 317 (17.8)	2948 (14.0)	4928 (18.2)	7441 (18.2)	2960 (13.5)	2413 (11.5)	3606 (12.1)	2035 (14.0)	2808 (18.0)	4378 (50.7)	1181 (7.6)	828 (13.2)	4510 (12.8)	53 353 (16.0)
Missing	25 (<0.1)	264 (1.3)	256 (0.9)	1727 (4.2)	2067 (9.4)	10111 (48.0)	8163 (27.4)	355 (2.5)	735 (4.7)	305 (3.5)	8504 (54.6)	2492 (39.7)	7 (<0.1)	35 011 (10.5)
Age at menopause, y														
Premenopausal	37 093 (49.5)	9538 (45.3)	8900 (32.9)	5116 (12.5)	8598 (39.3)	3773 (17.9)	11 897 (39 8)	6156 (42.5)	4340 (27.8)	285 (3.3)	5934 (38.1)	752 (12.0)	9388 (26.6)	111 770 (33.6)
<45	6266 (8.4)	1724 (8.2)	2449 (9.0)	2719 (6.7)	1959 (8.9)	1820 (8.7)	2416 (8.1)	751 (5.2)	1133 (7.2)	1065 (12.2)	709 (4.5)	1353 (21.6)	3125 (8.9)	27 479 (8.3)
45-49	16 394 (21. 9)	4153 (19.7)	5800 (21.4)	8993 (22.0)	3518 (16.1)	4189 (19.9)	5036 (16.9)	2226 (15.4)	2582 (16.5)	1494 (17.3)	2240 (14.4)	1755 (28.0)	7944 (22.5)	66 324 (19.9)
50-54	13 218 (17.6)	4993 (23.7)	8396 (31.0)	13 901 (34.0)	4512 (20.6)	6312 (30.0)	5382 (18.0)	4431 (30.6)	3039 (19.4)	1901 (22.0)	3732 (23.9)	1893 (30.2)	12511 (35.5)	84 221 (25.3)
≥55	1439 (1.9)	296 (1.4)	929 (3.4)	1561 (3.8)	423 (1.9)	820 (3.9)	557 (1.9)	441 (3.0)	314 (2.0)	417 (4.8)	723 (4.6)	393 (6.3)	1936 (5.5)	10 249 (3.1)
Missing	482 (0.6)	369 (1.8)	620 (2.3)	8546 (20.9)	2893 (13.2)	4129 (19.6)	4598 (15.4)	486 (3.3)	4227 (27.0)	3488 (40.4)	2248 (14.4)	131 (2.1)	365 (1.0)	32 582 (9.8)

Abbreviations: 3P, 3-Prefecture; BMI, body mass index (calculated as weight in kilograms divided by height in meters squared; HT, hormone therapy; JACC, Japan Collaborative Cohort Study; JPHC, Japan Public Health Center-Based Prospective Study; KMCC, Korean Multicenter Cancer Cohort Study; KNCC, Korean National Cancer Center Cohort Study; LSS, Life Span Study; NA, not applicable; SCHS, Singapore Chinese Health Study; SWHS, Shanghai Women’s Health Study.

The results pertaining to associations between reproductive factors and the risk of endometrial cancer are shown in Table 2. Participants who reported ever being pregnant showed a significantly lower risk of endometrial cancer than the nulliparous group (HR, 0.54; 95% CI, 0.48-0.67). An increased number of deliveries was associated with a decreased risk of endometrial cancer. Compared with nulliparous women, the HRs for women with 1 to 2, 3 to 4, and 5 or more deliveries were 0.54 (95% CI, 0.42-0.70), 0.50 (95% CI, 0.38-0.64), and 0.31 (95% CI, 0.22-0.45), respectively (*P *for trend < .001). Among parous women, when the lowest category of number of deliveries (1-2) was set as a reference, the HRs for the highest category (≥5 deliveries) was 0.57 (95% CI, 0.42-0.78) (*P *for trend = .003). The results adjusted for age as a categorical variable were similar and are shown in eTable 1 in Supplement 1.

**Table 2.** Association Between Reproductive Factors and Endometrial Cancer Risk Among Prospective Cohorts in the Asia Cohort Consortium

Factor	No. of cases	No. of participants	Age-adjusted HR (95% CI)^a^	Multivariable-adjusted (with LSS)^b^	Multivariable-adjusted (excluding LSS)^b^
Pregnancy					
Never	103	23 035	1 [Reference]	1 [Reference]	1 [Reference]
Ever	902	309 590	0.58 (0.47-0.71)	0.54 (0.48-0.67)	0.51 (0.40-0.65)
No. of deliveries					
0	72	14 298	1 [Reference]	NA	1 [Reference]
1-2	496	152 056	0.58 (0.45-0.75)	NA	0.54 (0.42-0.70)
3-4	276	101 606	0.56 (0.43-0.72)	NA	0.50 (0.38-0.64)
≥5	54	34 166	0.37 (0.26-0.53)	NA	0.31 (0.22-0.45)
*P* for trend	NA	NA	<.001	NA	<.001
No. of deliveries among parous women					
1-2	496	152 056	11 [Reference]	NA	1 [Reference]
3-4	276	101 606	0.94 (0.80-1.10)	NA	0.90 (0.76-1.06)
≥5	54	34 166	0.61 (0.45-0.83)	NA	0.57 (0.42-0.78)
*P* for trend	NA	NA	.01	NA	.003
Age at first delivery among parous women, y^c^					
≤20	97	34 418	1 [Reference]	1 [Reference]	1 [Reference]
21-25	451	152 016	1.05 (0.84-1.31)	1.11 (0.89-1.39)	1.05 (0.82-1.33)
26-30	278	95 634	0.91 (0.71-1.16)	0.99 (0.77-1.26)	0.94 (0.73-1.21)
≥31	63	21 169	0.91 (0.66-1.26)	0.99 (0.72-1.37)	0.91 (0.65-1.28)
*P* for trend	NA	NA	.12	.20	.20
Age at menarche, y					
<13	93	23 751	1 [Reference]	1 [Reference]	1 [Reference]
13-14	402	109 950	0.95 (0.76-1.20)	0.99 (0.79-1.24)	0.99 (0.78-1.25)
15-16	336	110 560	0.84 (0.67-1.07)	0.89 (0.70-1.13)	0.91 (0.71-1.16)
≥17	105	53 353	0.60 (0.45-0.80)	0.64 (0.48-0.86)	0.66 (0.49-0.89)
*P* for trend	NA	NA	<.001	<.001	.002
Age at menopause among postmenopausal women, y					
<45	39	27 479	1 [Reference]	1 [Reference]	1 [Reference]
45-49	122	66 324	1.32 (0.92-1.89)	1.33 (0.92-1.91)	1.36 (0.93-2.00)
50-54	235	84 221	2.15 (1.52-3.04)	2.14 (1.51-3.02)	2.12 (1.46-3.06)
≥55	35	10 249	2.92 (1.82-4.67)	2.84 (1.78-4.55)	2.90 (1.77-4.75)
*P* for trend	NA	NA	<.001	<.001	<.001
HT use^d^					
Never	586	169 003	1 [Reference]	NA	1 [Reference]
Ever	17	10 246	0.57 (0.35-0.93)	NA	0.62 (0.38-1.01)
Breastfeeding^d^					
Never	55	15 189	1 [Reference]	NA	1 [Reference]
Ever	246	97 195	0.77 (0.57-1.04)	NA	0.76 (0.56-1.02)

Abbreviations: HR, hazard ratio; HT, hormone therapy; LSS, Life Span Study; NA, not applicable.

^a^ Adjusted for age at baseline. HRs were calculated using the Cox proportional hazard frailty model (with random effects).

^b^ Adjusted for age at baseline, body mass index, age at menarche, age at menopause (including premenopausal), current smoking, current drinking, parity, HT use, and breastfeeding. HRs were calculated using the Cox proportional hazard frailty model (with random effects).

^c^ Japan Collaborative Cohort Study and LSS included questions about the age at first pregnancy (not delivery).

^d^ Model for HT use included 8 cohorts and that for breastfeeding included 7 cohorts.

Older age at menarche and younger age at menopause were significantly associated with a lower risk of endometrial cancer. When the youngest categories of age at menarche (<13 years) and menopause (<45 years) were defined as references, the HR of the highest age at menarche (≥17 years) was 0.64 (95% CI, 0.48-0.86; *P *for trend = .004) and that of menopause (≥55 years) was 2.84 (95% CI, 1.78-4.55; *P *for trend < .001). Age at first delivery, HT use, and breastfeeding were not associated with endometrial cancer risk, although point estimates of the HR for HT use and ever breastfeeding were less than 1.0. We examined the influence of one study by excluding each study, but the results did not change largely (eTable 2 in Supplement 1). Moreover, the results did not change significantly when missing values were treated as a single category.

The Figure shows the results stratified by BMI, menopausal status, and parity, and the detailed results are shown in eTable 3 in Supplement 1. Most results were similar to those obtained in the main analyses. Because the number of cancer cases became small due to stratification, the associations of pregnancy among postmenopausal women and the number of deliveries among parous women with lower BMI in the premenopausal group were not statistically significant. In addition, because the number of deliveries was not asked in LSS, we examined the association excluding LSS (eTable 4 in Supplement 1). Although the results were not largely changed, the results for age at menarche were no longer statistically significant.

**Figure.**
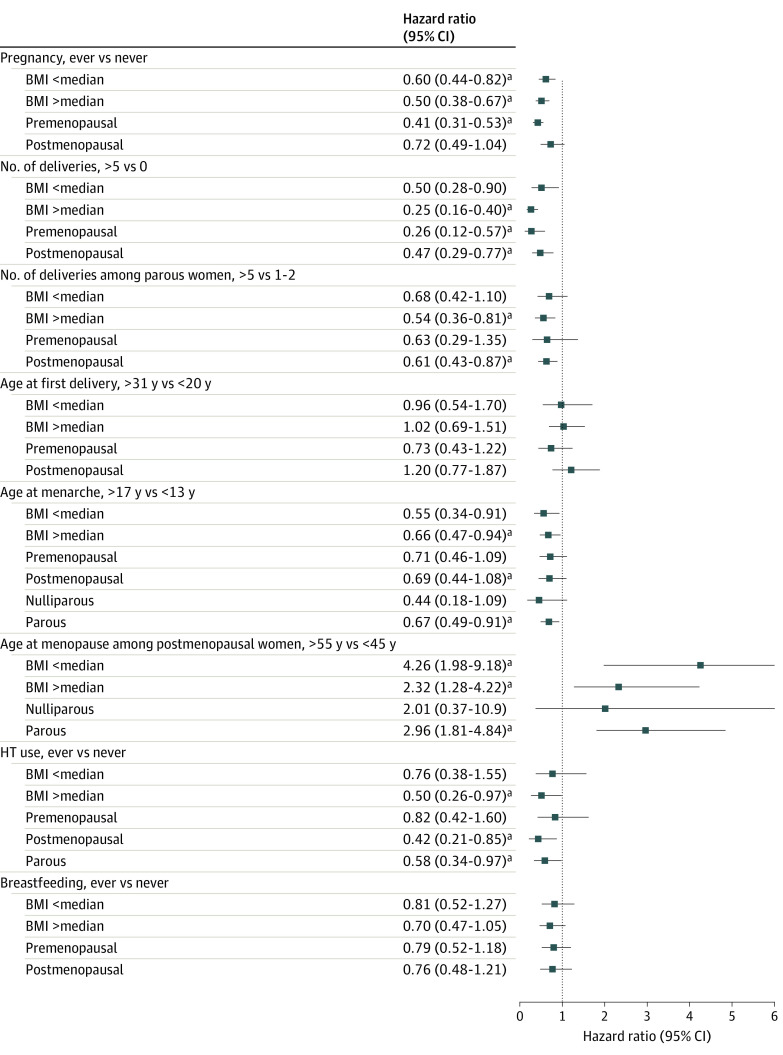
Stratified Analyses Between Reproductive Factors and Endometrial Cancer Risk by Body Mass Index (BMI), Menopausal Status, and Parous Status The model for hormone therapy (HT) use included 8 cohorts and that for breastfeeding included 7 cohorts. Detailed results are in eTable 3 in Supplement 1. Models were adjusted for age at baseline, BMI, age at menarche, age at menopause (including premenopause), current smoking, current drinking, parity, HT use, and breastfeeding, except for the stratification variable. Japan Collaborative Cohort Study and Life Span Study included a question about the age at first pregnancy (not delivery). ^a^Significant (*P* < .05) trend of categories of each reproductive factor.

## Discussion

In this pooled analysis comprising 332 625 participants from 13 cohort studies, parity, a greater number of deliveries, late menarche, and early menopause were associated with a lower risk of endometrial cancer among Asian women. Because there are a limited number of prospective reports owing to the low incidence of endometrial cancer, this report provides evidence regarding reproductive factors and endometrial cancer in Asian countries.

The results regarding multiparity, age at menarche, and age at menopause in this study were consistent with previously published evidence. In an umbrella review,^16^ parity showed strong evidence, delay in achieving menarche showed suggestive evidence, and prolonged breastfeeding showed weak evidence of a lower risk of endometrial cancer. Although that review included 171 meta-analyses, only 6 focused on the reproductive factors investigated in this study. Among more than 100 individual studies included in these 6 meta-analyses, 7 were from Asian countries, and only 2 were prospective. Regarding other reproductive factors investigated in other meta-analyses, an increase in parity and early menopause showed an association with a lower risk of endometrial cancer, similar to the results of our study.^14,15^ One systematic review^35^ showed that breastfeeding was significantly associated with a reduced relative risk (RR) of endometrial cancer, particularly in North America (6 studies; RR, 0.87; 95% CI, 0.79-0.95) but not in Asia (4 studies; RR, 0.58; 95% CI, 0.31-1.07).^35^ Consistent with these results, we did not find a significant association between breastfeeding and endometrial cancer. Breastfeeding, which suppresses ovulation, may reduce the risk of endometrial cancer. Although our pooled results can be regarded as including the maximum number of cases possible from Asian prospective studies, further studies including information on the duration of breastfeeding and a larger number of cancer cases might be required to establish evidence for the association between breastfeeding and endometrial cancer.

Given that the age at menarche is likely to be higher in Asian populations than in European and US populations,^36^ a different category of menarche age was used in Asian studies. We included cohorts in which data pertaining to age were acquired categorically, with the lowest and highest categories being the ages of 13 years or younger and 17 years or older, respectively, whereas studies conducted in Europe and the United States applied the lowest and highest age categories of 11 years and 14 or 15 years, respectively.^5,6,11^ On the other hand, the category of age at menopause was similar, although natural menopause has been shown to occur at a later age among Japanese Americans than in other ethnic groups.^37^ Some Western studies applied the lowest age category of 50 years^6,38^; however, an age category of 45 years or younger was used overall, regardless of the country.^5,7,37^ Although the categories varied slightly among studies and countries, the associations between age at menarche and menopause and endometrial cancer were consistent.

Moreover, age at menarche and menopause are influenced by BMI.^39^ The consistent results from Asian countries with lower BMI (mean BMI, 22.0-24.6 in our analysis) are worth reporting to broaden the evidence. In a dose-response meta-analysis of BMI,^40^ the risk of endometrial cancer increased more obviously when BMI was 25 or greater. In our analysis, the *P* value for the interaction between the higher and lower BMI groups was not significant, and the trend of age at menopause in the low BMI group was significant (median BMI, approximately 23). While some reproductive factors, such as the number of deliveries, did not show significant associations in the low-BMI group, it is possible that protective effects of these factors may occur in higher-BMI groups. However, the limited number of cases in the low-BMI group indicates the need for further studies with larger numbers of participants from lower-BMI populations. The presence of unopposed estrogen underlies the association between selected reproductive factors and the risk of endometrial cancer.^9,10,41^ Excess estrogen causes endometrial cell proliferation and is a risk factor for endometrial cancer.^42^ Parity, menarche, and menopause cause changes in estrogen and progesterone levels. For example, parous women have lower estrogen levels than nulliparous women.^43^ In line with this, higher age at last birth has been proposed to be associated with a lower risk of endometrial cancer because decreased estrogen levels accompanying pregnancy among women approaching menopause may have a protective effect against cancer. Moreover, OC use might reduce the cancer risk, and HT (especially estrogen therapy) is a potential risk factor for endometrial cancer.^44,45^ However, in the present study, age at last birth, OC use, and type of HT could not be investigated as exposure or confounding factors because the age at last birth and type of HT were not included in the questionnaires, and OC and HT use were uncommon in Asian countries, particularly in the 1990s. According to data from the United Nations in 2019, the estimated prevalence rates of OC use were 2.9% in Japan and 3.3% in Korea, compared with 15.1% in North America and 19.1% in European countries.^46^ Regarding HT use, the total prevalence reported in surveys of nurses in Japan was 13.8%, which was lower than that in non-Asian countries.^47^ Although most of the participating cohorts did not include information on the type of HT, a Korean study^48^ found that the proportion of HT use among women older than 40 years was 7.8%, and the rate of estrogen therapy was 3 times higher than that of estrogen-plus-progesterone therapy in 2002.^48^ In the future, such a study including HT type may be required because the lifestyle in Asian countries may be westernized.

### Strengths and Limitations

This study has strengths. To our knowledge, it is the largest pooled analysis of Asian prospective studies with a large number of cases and long follow-up durations. Although generalizability of results was limited to Asians, individual data from multiple sites in Asia were collected and analyzed.

However, our study also has several limitations. First, each cohort used a different questionnaire, and questions regarding reproductive factors differed slightly among the participating cohorts. Although we checked each questionnaire, and data cleaning was performed by a working group, JACC and LSS included questions about the age at first pregnancy, and categorical variables were applied to include all possible cohorts. Second, not all cohorts were asked about all reproductive factors investigated in this study, such as a history of hysterectomy, duration of breastfeeding, type of HT, and number of deliveries in LSS. Histological cancer types or types I and II classifications were not considered in this study because morphological information was obtained in 5 cohorts. As obesity and reproductive factors might be influenced more by type II tumors,^49^ further studies including endometrial cancer types are essential. Moreover, we did not collect information on the history of cancer other than endometrial cancer in this study, and participants with a history of cancer other than endometrial cancer were not excluded from analyses. Third, confounding factors other than age, country, smoking status, and drinking status were not included in the model. Physical activity, family history of endometrial cancer, and education level might be important confounding factors but could not be adjusted for in this study due to lack of data. Although further studies with full adjustment are ideal, we observed an HR trend that is consistent with the existing evidence, and these confounding factors may not have changed the results. Fourth, because we collected the data on reproductive factors at baseline, misclassification may have occurred, especially for factors such as menopausal status that change with time. Harmonization of time-varying data should be considered in future studies.

## Conclusions

In conclusion, among 1005 endometrial cancer cases recorded in 13 Asian cohort studies comprising 332 625 women, late menarche, early menopause, and a higher number of deliveries were significantly associated with a lower risk of endometrial cancer. These results are consistent with previous evidence emanating mainly from non-Asian countries. Breastfeeding was not significantly associated with endometrial cancer in this study. Since Asian countries are characterized by a low incidence of endometrial cancer, this study contributes to expanding the generalizability of evidence on the risk and protective factors for endometrial cancer.

## Notes

Supplement 1. **eTable 1.** Association Between Reproductive Factors and Endometrial Cancer Risk Including Models Adjusted for Age as Categorical Variable **eTable 2.** Association Between Reproductive Factors and Endometrial Cancer Risk Excluding One Cohort Study **eTable 3.** Subgroup Analyses of the Association Between Reproductive Factors and Endometrial Cancer Risk **eTable 4.** Subgroup Analyses of the Association Between Reproductive Factors and Endometrial Cancer Risk Excluding LSS **eAppendix.** Supplemental Material

Supplement 2.
Data Sharing Statement
