# Supplementary material for: Reproductive Factors and Endometrial Cancer Risk Among Women
Source: JAMA Netw Open. 2023 Sep 5;6(9):e2332296. doi: 10.1001/jamanetworkopen.2023.32296 (PMC10481237; doi:10.1001/jamanetworkopen.2023.32296)
Supplement: Supplement 1. — eTable 1. Association Between Reproductive Factors and Endometrial Cancer Risk Including Models Adjusted for Age as Categorical Variable eTable 2. Association Between Reproductive Factors and Endometrial Cancer Risk Excluding One Cohort Study eTable 3. Subgroup Analyses of the Association Between Reproductive Factors and Endometrial Cancer Risk eTable 4. Subgroup Analyses of the Association Between Reproductive Factors and Endometrial Cancer Risk Excluding LSS eAppendix. Supplemental Material [file jamanetwopen-e2332296-s001.pdf]

Supplemental Online Content

Katagiri R, Iwasaki M, Abe SK, et al. Reproductive factors and endometrial cancer risk among women. *JAMA Netw Open.* 2023;6(9):e2332296. doi:10.1001/jamanetworkopen.2023.32296

eTable 1. Association Between Reproductive Factors and Endometrial Cancer Risk Including Models Adjusted for Age as Categorical Variable

eTable 2. Association Between Reproductive Factors and Endometrial Cancer Risk Excluding One Cohort Study

eTable 3. Subgroup Analyses of the Association Between Reproductive Factors and Endometrial Cancer Risk

eTable 4. Subgroup Analyses of the Association Between Reproductive Factors and Endometrial Cancer Risk Excluding LSS

eAppendix. Supplemental Material

This supplemental material has been provided by the authors to give readers additional information about their work.

© 2023 Katagiri R et al. *JAMA Network Open.*

eTable 1. Association Between Reproductive Factors and Endometrial Cancer Risk Including Models Adjusted for Age as Categorical Variable

	No. of cases	No. of participants	Age-adjusted HR[1] (95% CI)	Age-adjusted HR[2] (95% CI)	Multivariable-adjusted[3] (with LSS)	Multivariable-adjusted[4] (with LSS)	Multivariable-adjusted[3] (excluding LSS)	Multivariable-adjusted[4] (excluding LSS)
Reproductive factors								
Pregnancy								
Never	103	23035	1.00 (reference)	1.00 (reference)	1.00 (reference)	1.00 (reference)	1.00 (reference)	1.00 (reference)
Ever	902	309590	0.58 (0.47–0.71)	0.57 (0.46–0.70)	0.54 (0.48–0.67)	0.54 (0.43–0.66)	0.51 (0.40–0.65)	0.51 (0.40–0.65)
No. of deliveries								
0	72	14298	1.00 (reference)	1.00 (reference)	-	-	1.00 (reference)	1.00 (reference)
1–2	496	152056	0.58 (0.45–0.75)	0.58 (0.45–0.75)	-	-	0.54 (0.42–0.70)	0.55 (0.42–0.70)
3–4	276	101606	0.56 (0.43–0.72)	0.55 (0.42–0.72)	-	-	0.50 (0.38–0.64)	0.50 (0.38–0.65)
≥5	54	34166	0.37 (0.26–0.53)	0.36 (0.25–0.52)	-	-	0.31 (0.22–0.45)	0.31 (0.22–0.45)
p-value for trend			<0.001	<0.001			<0.001	<0.001
No. of deliveries (among parous women)								
1–2	496	152056	1.00 (reference)	1.00 (reference)	-	-	1.00 (reference)	1.00 (reference)
3–4	276	101606	0.94 (0.80–1.10)	0.93 (0.79–1.09)	-	-	0.90 (0.76–1.06)	0.89 (0.76–1.04)
≥5	54	34166	0.61 (0.45–0.83)	0.60 (0.44–0.81)	-	-	0.57 (0.42–0.78)	0.56 (0.41–0.76)
p-value for trend			0.01	0.008			0.003	0.001
Age at first delivery (among parous women), years								
≤20	97	34418	1.00 (reference)	1.00 (reference)	1.00 (reference)	1.00 (reference)	1.00 (reference)	1.00 (reference)
21–25	451	152016	1.05 (0.84–1.31)	1.05 (0.84–1.31)	1.11 (0.89–1.39)	1.10 (0.88–1.38)	1.05 (0.82–1.33)	1.04 (0.81–1.32)
26–30	278	95634	0.91 (0.71–1.16)	0.91 (0.71–1.16)	0.99 (0.77–1.26)	0.97 (0.76–1.24)	0.94 (0.73–1.21)	0.93 (0.72–1.20)
≥31	63	21169	0.91 (0.66–1.26)	0.91 (0.66–1.26)	0.99 (0.72–1.37)	0.97 (0.70–1.34)	0.91 (0.65–1.28)	0.88 (0.63–1.24)
p-value for trend			0.12	0.14	0.20	0.26	0.20	0.17
Age at menarche, years								
<13	93	23751	1.00 (reference)	1.00 (reference)	1.00 (reference)	1.00 (reference)	1.00 (reference)	1.00 (reference)
13–14	402	109950	0.95 (0.76–1.20)	0.95 (0.76–1.21)	0.99 (0.79–1.24)	0.97 (0.78–1.22)	0.99 (0.78–1.25)	0.99 (0.78–1.26)
15–16	336	110560	0.84 (0.67–1.07)	0.84 (0.66–1.06)	0.89 (0.70–1.13)	0.88 (0.69–1.11)	0.91 (0.71–1.16)	0.91 (0.71–1.16)
≥17	105	53353	0.60 (0.45–0.80)	0.59 (0.44–0.79)	0.64 (0.48–0.86)	0.64 (0.48–0.86)	0.66 (0.49–0.89)	0.64 (0.47–0.87)
p-value for trend			<0.001	<0.001	<0.001	<0.001	0.002	0.001
Age at menopause, years (categorical, among postmenopausal women)								
<45	39	27479	1.00 (reference)	1.00 (reference)	1.00 (reference)	1.00 (reference)	1.00 (reference)	1.00 (reference)
45–49	122	66324	1.32 (0.92–1.89)	1.33 (0.92–1.91)	1.33 (0.92–1.91)	1.30 (0.90–1.87)	1.36 (0.93–2.00)	1.35 (0.92–1.99)

© 2023 Katagiri R et al. *JAMA Network Open.*

50–54	235	84221	2.15 (1.52–3.04)	2.14 (1.51–3.02)	2.14 (1.51–3.02)	2.07 (1.47–2.93)	2.12 (1.46–3.06)	2.10 (1.45–3.03)
≥55	35	10249	2.92 (1.82–4.67)	2.77 (1.74–4.43)	2.84 (1.78–4.55)	2.70 (1.69–4.33)	2.90 (1.77–4.75)	2.82 (1.72–4.62)
p-value for trend			<0.001	<0.001	<0.001	<0.001	<0.001	<0.001
HRT use								
Never	586	169003	1.00 (reference)	1.00 (reference)	-	-	1.00 (reference)	1.00 (reference)
Ever	17	10246	0.57 (0.35–0.93)	0.57 (0.35–0.93)	-	-	0.62 (0.38–1.01)	0.63 (0.39–1.03)
Breastfeeding								
Never	55	15189	1.00 (reference)	1.00 (reference)	-	-	1.00 (reference)	1.00 (reference)
Ever	246	97195	0.77 (0.57–1.04)	0.79 (0.58–1.06)	-	-	0.76 (0.56–1.02)	0.77 (0.57–1.04)

Values are hazard ratios (95% confidence interval). Hazard ratios were calculated using the Cox proportional hazards frailty model (random effects).
1. Models were adjusted for age at the baseline (continuous), BMI, age at menarche, age at menopause (including premenopause), current smoking, current drinking, parity, HRT use, and breastfeeding.
2. Models were adjusted for age at the baseline (category: ≤46, 47-54, 55-62, and ≥63), BMI, age at menarche, age at menopause (including premenopause), current smoking, current drinking, parity, HRT use, and breastfeeding.

© 2023 Katagiri R et al. *JAMA Network Open*.

eTable 2. Association Between Reproductive Factors and Endometrial Cancer Risk Excluding One Cohort Study

	Excluded cohort study												
	SWHS	JPHC1	JPHC2	JACC	Miyagi	Ohsaki	LSS	Takayama	3-Pref Miyagi	KMCC	KNCC	Namwon	SCHS
Pregnancy													
Never	1.00 (reference)	1.00 (reference)	1.00 (reference)	1.00 (reference)	1.00 (reference)	1.00 (reference)	1.00 (reference)	1.00 (reference)	1.00 (reference)	1.00 (reference)	1.00 (reference)	1.00 (reference)	1.00 (reference)
Ever	0.52 (0.41–0.66)	0.54 (0.43–0.68)	0.52 (0.41–0.64)	0.48 (0.39–0.60)	0.49 (0.40–0.61)	0.50 (0.41–0.62)	0.51 (0.40–0.65)	0.50 (0.41–0.63)	0.52 (0.41–0.66)	0.50 (0.40–0.61)	0.50 (0.40–0.63)	0.51 (0.41–0.63)	0.51 (0.41–0.63)
No. of deliveries													
0	1.00 (reference)	1.00 (reference)	1.00 (reference)	1.00 (reference)	1.00 (reference)	1.00 (reference)	NA	1.00 (reference)	1.00 (reference)	1.00 (reference)	1.00 (reference)	1.00 (reference)	1.00 (reference)
1–2	0.56 (0.43–0.74)	0.59 (0.44–0.79)	0.56 (0.43–0.74)	0.50 (0.39–0.65)	0.53 (0.41–0.69)	0.55 (0.42–0.71)	NA	0.55 (0.42–0.71)	0.59 (0.45–0.76)	0.53 (0.41–0.68)	0.55 (0.42–0.71)	0.55 (0.42–0.71)	0.52 (0.39–0.69)
3–4	0.49 (0.37–0.65)	0.52 (0.38–0.70)	0.49 (0.37–0.65)	0.46 (0.35–0.60)	0.48 (0.37–0.63)	0.47 (0.36–0.62)	NA	0.48 (0.37–0.63)	0.52 (0.40–0.69)	0.48 (0.37–0.62)	0.49 (0.37–0.64)	0.48 (0.37–0.63)	0.46 (0.35–0.62)
≥5	0.30 (0.23–0.45)	0.31 (0.20–0.46)	0.30 (0.20–0.45)	0.29 (0.20–0.42)	0.29 (0.20–0.42)	0.29 (0.20–0.43)	NA	0.30 (0.21–0.44)	0.33 (0.22–0.48)	0.31 (0.21–0.45)	0.31 (0.21–0.45)	0.32 (0.22–0.46)	0.28 (0.18–0.44)
p-value for trend	<0.001	<0.001	<0.001	<0.001	<0.001	<0.001	NA	<0.001	<0.001	<0.001	<0.001	<0.001	<0.001
No. of deliveries (among parous women)													
1–2	1.00 (reference)	1.00 (reference)	1.00 (reference)	1.00 (reference)	1.00 (reference)	1.00 (reference)	NA	1.00 (reference)	1.00 (reference)	1.00 (reference)	1.00 (reference)	1.00 (reference)	1.00 (reference)
3–4	0.92 (0.77–1.09)	0.88 (0.74–1.05)	0.85 (0.72–1.01)	0.92 (0.77–1.09)	0.89 (0.74–1.06)	0.84 (0.71–1.00)	NA	0.87 (0.73–1.02)	0.88 (0.75–1.04)	0.88 (0.75–1.04)	0.89 (0.75–1.05)	0.88 (0.74–1.03)	0.88 (0.74–1.05)
≥5	0.58 (0.42–0.80)	0.60 (0.44–0.84)	0.54 (0.39–0.75)	0.58 (0.42–0.80)	0.54 (0.39–0.74)	0.53 (0.38–0.74)	NA	0.55 (0.40–0.76)	0.56 (0.41–0.77)	0.58 (0.42–0.79)	0.57 (0.41–0.78)	0.59 (0.43–0.80)	0.55 (0.37–0.82)
p-value for trend	0.002	0.006	<0.001	0.007	0.001	0.003	NA	<0.001	0.002	0.002	0.002	0.002	0.008
Age at first delivery[1] (among parous women), years													
≤20	1.00 (reference)	1.00 (reference)	1.00 (reference)	1.00 (reference)	1.00 (reference)	1.00 (reference)	1.00 (reference)	1.00 (reference)	1.00 (reference)	1.00 (reference)	1.00 (reference)	1.00 (reference)	1.00 (reference)
21–25	1.13 (0.87–1.48)	1.05 (0.83–1.32)	1.16 (0.92–1.46)	1.12 (0.89–1.31)	1.09 (0.86–1.38)	1.18 (0.94–1.50	1.05 (0.82–1.33)	1.12 (0.89–1.41)	1.11 (0.88–1.39)	1.10 (0.88–1.38)	1.15 (0.91–1.44)	1.13 (0.90–1.42)	1.19 (0.92–1.54)
26–30	0.94 (0.73–1.21)	0.98 (0.76–1.26)	1.03 (0.80–1.32)	0.95 (0.74–1.22)	0.99 (0.77–1.28)	0.91 (0.81–1.34)	0.94 (0.73–1.21)	1.00 (0.78–1.28)	0.99 (0.77–1.26)	0.96 (0.75–1.23)	1.01 (0.79–1.29)	1.00 (0.79–1.28)	1.07 (0.81–1.41)
≥31	0.91 (0.65–1.28)	1.00 (0.72–1.40)	1.00 (0.72–1.41)	1.00 (0.72–1.39)	1.02 (0.74–1.43)	1.05 (0.76–1.47)	0.91 (0.65–1.28)	1.04 (0.74–1.44)	0.98 (0.71–1.36)	0.99 (0.71–1.37)	1.04 (0.75–1.44)	1.02 (0.74–1.41)	1.02 (0.74–1.41)
p-value for trend	0.29	0.63	0.40	0.24	0.20	0.44	0.20	0.49	0.33	0.27	0.43	0.41	0.75
Age at menarche,													

© 2023 Katagiri R et al. *JAMA Network Open.*

years													
<13	1.00 (reference)	1.00 (reference)	1.00 (reference)	1.00 (reference)	1.00 (reference)	1.00 (reference)	1.00 (reference)	1.00 (reference)	1.00 (reference)	1.00 (reference)	1.00 (reference)	1.00 (reference)	1.00 (reference)
13–14	0.93 (0.72–1.21)	0.97 (0.77–1.23)	1.00 (0.79–1.23)	0.98 (0.78–1.24)	1.02 (0.81–1.30)	0.97 (0.77–1.22)	0.99 (0.78–1.26)	1.03 (0.81–1.31)	0.99 (0.79–1.25)	1.00 (0.80–1.26)	0.99 (0.79–1.24)	0.98 (0.78–1.24)	0.98 (0.76–1.27)
15–16	0.84 (0.64–1.11)	0.83 (0.65–1.06)	0.89 (0.70-1.14)	0.86 (0.68-1.10)	0.91 (0.71-1.17)	0.88 (0.70–1.12)	0.91 (0.71–1.16)	0.94 (0.73-1.20)	0.89 (0.70–1.13)	0.90 (0.71-1.13)	0.89 (0.70–1.13)	0.89 (0.70–1.12)	0.93 (0.71–1.21)
≥17	0.57 (0.40–0.80)	0.59 (0.44–0.81)	0.67 (0.50–0.90)	0.62 (0.46–0.84)	0.63 (0.46–0.86)	0.65 (0.48–0.86)	0.66 (0.49–0.89)	0.68 (0.50–0.91)	0.66 (0.50–0.89)	0.66 (0.49–0.88)	0.64 (0.47–0.85)	0.63 (0.47–0.84)	0.69 (0.50–0.95)
p-value for trend	<0.001	<0.001	0.002	<0.001	<0.001	<0.001	0.002	0.002	<0.001	<0.001	<0.001	<0.001	0.006
Age at menopause, years (categorical, among postmenopausal women)													
<45	1.00 (reference)	1.00 (reference)	1.00 (reference)	1.00 (reference)	1.00 (reference)	1.00 (reference)	1.00 (reference)	1.00 (reference)	1.00 (reference)	1.00 (reference)	1.00 (reference)	1.00 (reference)	1.00 (reference)
45–49	1.15 (0.75–1.75)	1.35 (0.92–1.98)	1.36 (0.93–1.99)	1.35 (0.93–1.98)	1.31 (0.90–1.90)	1.34 (0.92–1.94)	1.36 (0.93–2.00)	1.34 (0.93–1.94)	1.27 (0.88–1.83)	1.29 (0.90–1.86)	1.37 (0.95–1.97)	1.35 (0.93–1.94)	1.32 (0.89–1.96)
50–54	1.99 (1.34–3.01)	2.09 (1.45–3.01)	2.24 (1.57–3.24)	2.18 (1.52–3.13)	2.02 (1.41–2.90)	2.13 (1.49–3.03)	2.12 (1.46–3.06)	2.14 (1.51–3.04)	2.08 (1.47-2.94)	2.11 (1.50-2.99)	2.16 (1.52–3.07)	2.13 (1.50–3.03)	2.20 (1.52–3.21)
≥55	2.49 (1.45–4.31)	2.90 (1.79–4.70)	3.20 (1.97–5.17)	3.00 (1.85–4.88)	2.34 (1.41–3.88)	3.14 (1.95–5.07)	2.90 (1.77–4.75)	2.85 (1.77–4.59)	2.74 (1.77–4.53)	2.83 (1.77–4.53)	2.98 (1.86–4.78)	2.92 (1.82–4.68)	2.50 (1.46–4.28)
p-value for trend	<0.001	<0.001	<0.001	<0.001	<0.001	<0.001	<0.001	<0.001	<0.001	<0.001	<0.001	<0.001	<0.001
HRT use													
Never	1.00 (reference)	NA	NA	NA	1.00 (reference)	1.00 (reference)	NA	1.00 (reference)	NA	1.00 (reference)	1.00 (reference)	1.00 (reference)	1.00 (reference)
Ever	0. 65 (0.35–1.19)	NA	NA	NA	0. 55 (0.31–0.99)	0. 69 (0.42–1.13)	NA	0. 57 (0.34–0.95)	NA	0. 58 (0.35–0.95)	0. 67 (0.42–1.09)	0. 63 (0.39–1.02)	0. 65 (0.39–1.09)
Breastfeeding													
Never	NA	1.00 (reference)	1.00 (reference)	NA	1.00 (reference)	1.00 (reference)	NA	NA	NA	1.00 (reference)	1.00 (reference)	1.00 (reference)	NA
Ever	NA	0.87 (0.60–1.25)	0. 68 (0.50–0.94)	NA	0. 74 (0.51–1.05)	0. 77 (0.55–1.06)	NA	NA	NA	0. 77 (0.57–1.03)	0. 74 (0.54–1.01)	0. 76 (0.56–1.02)	NA

Values are hazard ratios (95% confidence interval). We excluded one cohort and included all the other cohorts (we excluded LSS from all analyses about the number of pregnancy).

Abbreviations: SWHS, Shanghai Women's Health Study; JPHC, Japan Public Health Center-based Prospective Study; JACC, Japan Collaborative Cohort Study; Miyagi, Miyagi Cohort Study; Ohsaki, Ohsaki National Health Insurance Cohort Study; LSS, Life Span Study; Takayama, Takayama Cohort Study; 3-Pref Miyagi, Three-Prefecture Cohort Study Miyagi; KMCC, Korean Multi-center Cancer Cohort Study; SCHS, Singapore Chinese Health Study; Namwon, Namwon Study; KNCC, Korean National Cancer Center Cohort study

© 2023 Katagiri R et al. *JAMA Network Open.*

eTable 3. Subgroup Analyses of the Association Between Reproductive Factors and Endometrial Cancer Risk

	No. of cases	BMI <median	BMI >median	No. of cases	Premenopausal	Postmenopausal	No. of cases	Nulliparous	Parous
Pregnancy		-	-		111783	207643		-	-
Never	49/54	1.00 (reference)	1.00 (reference)	62/35	1.00 (reference)	1.00 (reference)		-	-
Ever	333/569	0.60 (0.44–0.82)	0.50 (0.38–0.67)	431/445	0.41 (0.31–0.53)	0.72 (0.49–1.04)		-	-
p-value for interaction		0.37			0.02				
No. of deliveries (n)		-	-		99739	191307		-	-
0	30/42	1.00 (reference)	1.00 (reference)	45/26	1.00 (reference)	1.00 (reference)		-	-
1–2	187/309	0.56 (0.38–0.82)	0.53 (0.38–0.73)	294/190	0.40 (0.29–0.55)	0.77 (0.51–1.17)		-	-
3–4	97/179	0.57 (0.38–0.86)	0.46 (0.33–0.65)	93/179	0.36 (0.25–0.52)	0.72 (0.48–1.10)		-	-
≥5	20/34	0.50 (0.28–0.90)	0.25 (0.16–0.40)	7/47	0.26 (0.12–0.57)	0.47 (0.29–0.77)		-	-
p-value for trend		0.11	<0.001		<0.001	0.005		-	-
p-value for interaction		0.89			0.26				
No. of deliveries (among parous women)		-	-		95032	182372			
1–2	210/286	1.00 (reference)	1.00 (reference)	294/190	1.00 (reference)	1.00 (reference)		-	-
3–4	111/165	0.94 (0.73–1.20)	0.89 (0.72–1.10)	93/179	0.90 (0.69–1.16)	0.93 (0.75–1.15)		-	-
≥5	22/32	0.68 (0.42–1.10)	0.54 (0.36–0.81)	7/47	0.63 (0.29–1.35)	0.61 (0.43–0.87)		-	-
p-value for trend		0.20	0.009		0.21	0.02		-	-
p-value for interaction		0.58			0.40				
Age at first delivery[1], years		-	-		104081	188174			
≤20	27/70	1.00 (reference)	1.00 (reference)	28/67	1.00 (reference)	1.00 (reference)		-	-
21–25	181/270	1.24 (0.82–1.87)	1.03 (0.79–1.36)	194/245	0.96 (0.64–1.44)	1.14 (0.86–1.52)		-	-
26–30	119/159	1.15 (0.75–1.78)	0.89 (0.67–1.20)	174/94	0.87 (0.57–1.33)	0.90 (0.66–1.25)		-	-
≥31	22/41	0.96 (0.54–1.70)	1.02 (0.69–1.51)	34/28	0.73 (0.43–1.22)	1.20 (0.77–1.87)		-	-

© 2023 Katagiri R et al. *JAMA Network Open.*

p-value for trend		0.56	0.42		0.13	0.56		-	-
p-value for interaction		0.78			0.39				
Age at menarche, years		-	-		102152	185067		15688	281926
<13	33/60	1.00 (reference)	1.00 (reference)	62/27	1.00 (reference)	1.00 (reference)	15/78	1.00 (reference)	1.00 (reference)
13–14	147/255	0.95 (0.65–1.38)	0.99 (0.74–1.31)	224/168	0.96 (0.73–1.28)	1.14 (0.76–1.71)	42/360	0.81 (0.45–1.48)	1.02 (0.80–1.30)
15–16	135/201	0.96 (0.65–1.41)	0.82 (0.61–1.10)	149/178	0.91 (0.67–1.23)	0.96 (0.64–1.45)	28/308	0.65 (0.34–1.24)	0.93 (0.72–1.19)
≥17	33/72	0.55 (0.34–0.91)	0.66 (0.47–0.94)	34/70	0.71 (0.46–1.09)	0.69 (0.44–1.08)	8/97	0.44 (0.18–1.09)	0.67 (0.49–0.91)
p-value for trend		0.02	0.004		0.12	0.003		0.05	0.002
p-value for interaction		0.10			0.75			0.38	
Age at menopause (among postmenopausal women), years		-	-					12421	175852
<45	13/26	1.00 (reference)	1.00 (reference)		-	-	5/34	1.00 (reference)	1.00 (reference)
45–49	39/83	1.27 (0.68–2.39)	1.36 (0.87–2.11)		-	-	5/117	0.57 (0.16–1.97)	1.42 (0.97–2.08)
50–54	85/150	2.32 (1.28–4.21)	2.06 (1.35–3.14)		-	-	19/216	2.08 (0.76–5.71)	2.16 (1.50–3.13)
≥55	15/20	4.26 (1.98–9.18)	2.32 (1.28–4.22)		-	-	2/33	2.01 (0.37–10.9)	2.96 (1.81–4.84)
p-value for trend		<0.001	<0.001		-	-		0.12	<0.001
p-value for interaction		0.23						0.90	
HRT use[2]		-	-		64205	114379		-	-
Never	217/369	1.00 (reference)	1.00 (reference)	301/285	1.00 (reference)	1.00 (reference)	-/547	-	1.00 (reference)
Ever	8/9	0.76 (0.38–1.55)	0.50 (0.26–0.97)	9/8	0.82 (0.42–1.60)	0.42 (0.21–0.85)	-/15	-	0.58 (0.34–0.97)
p-value for interaction		0.62			0.26				
Breastfeeding[2]					34587	74409		-	-
Never	25/30	1.00 (reference)	1.00 (reference)	31/22	1.00 (reference)	1.00 (reference)		-	-
Ever	110/136	0.81 (0.52–1.27)	0.70 (0.47–1.05)	101/140	0.79 (0.52–1.18)	0.76 (0.48–1.21)		-	-
p-value for interaction		0.23			0.64				

Values are hazard ratios (95% confidence interval). Hazard ratios were calculated using the Cox proportional hazards frailty model (random effects). Models were adjusted for age at the baseline, BMI, age at menarche, age at menopause (including premenopause), current smoking, current drinking, parity, HRT use, and breastfeeding, except for the stratification variable.

© 2023 Katagiri R et al. *JAMA Network Open.*

[1] JACC and LSS included a question about the age at first pregnancy (not delivery).
[2] The model for HRT use included eight cohorts and that of breastfeeding included seven cohorts. As the number of cases in the nulliparous group for HRT use was too small, we did not perform an analysis.

© 2023 Katagiri R et al. *JAMA Network Open.*

eTable 4. Subgroup Analyses of the Association Between Reproductive Factors and Endometrial Cancer Risk Excluding LSS

	No. of cases	BMI <median	BMI >median	No. of cases	Premenopausal	Postmenopausal	No. of cases	Nulliparous	Parous
Pregnancy					99886	191732			
Never	29/43	1.00 (reference)	1.00 (reference)	45/26	1.00 (reference)	1.00 (reference)			
Ever	285/543	0.56 (0.38–0.83)	0.48 (0.35–0.66)	394/417	0.38（0.28–0.53)	0.65 (0.43–0.96)			
p-value for interaction		0.27			0.04				
Age at first delivery[1], years					94183	178030			
≤20	21/65	1.00 (reference)	1.00 (reference)	23/63	1.00 (reference)	1.00 (reference)			
21–25	148/254	1.12 (0.70–1.79)	1.01 (0.76–1.34)	170/226	0.94 (0.60–1.46)	1.05 (0.78–1.40)			
26–30	112/157	1.05 (0.65–1.70)	0.86 (0.64–1.17)	167/92	0.85 (0.54–1.35)	0.85 (0.61–1.18)			
≥31	19/39	0.81 (0.43–1.52)	0.95 (0.63–1.43)	33/25	0.73 (0.42–1.25)	1.06 (0.66–1.69)			
p-value for trend		0.36	0.27		0.16	0.36			
p-value for interaction		0.50			0.51				
Age at menarche, years					91760	174670			
<13	28/58	1.00 (reference)	1.00 (reference)	58/25	1.00 (reference)	1.00 (reference)	12/74	1.00 (reference)	1.00 (reference)
13–14	118/242	0.95 (0.63–1.43)	0.97 (0.73–1.29)	197/156	0.93 (0.69–1.29)	1.16 (0.76–1.78)	33/327	0.93 (0.48–1.82)	0.99 (0.77–1.28)
15–16	117/191	1.05 (0.68–1.60)	0.80 (0.59–1.08)	136/166	0.89 (0.65–1.22)	1.00 (0.65–1.53)	21/287	0.74 (0.35–1.54)	0.92 (0.71–1.20)
≥17	27/67	0.59 (0.34–1.02)	0.64 (0.45–0.93)	31/63	0.70 (0.45–1.10)	0.69 (0.43–1.11)	3/91	0.28 (0.08–1.04)	0.68 (0.49–0.93)
p-value for trend		0.09	0.003		0.14	0.004		0.05	0.005
p-value for interaction		0.05			0.76			0.44	
Age at menopause (among postmenopausal women), years								7924	166958
<45	10/24	1.00 (reference)	1.00 (reference)				2/32	1.00 (reference)	1.00 (reference)
45–49	34/81	1.38 (0.68–2.81)	1.36 (0.86–2.16)				5/110	1.46 (0.28–7.63)	1.34 (0.90–1.99)
50–54	76/143	2.49 (1.27–4.89)	2.01 (1.29–3.12)				15/204	4.56 (0.99–21.0)	2.01 (1.38–2.95)
≥55	14/19	4.87 (2.10–11.3)	2.29 (1.24–4.25)				2/31	6.09 (0.76–48.6)	2.75 (1.66–4.58)
p-value for trend		<0.001	<0.001					0.02	<0.001
p-value for interaction		0.26						0.88	

Values are hazard ratios (95% confidence interval). Analyses were conducted excluding LSS for the reproductive factors that originally included LSS.

© 2023 Katagiri R et al. *JAMA Network Open.*

eAppendix. Supplemental Material

In 2020, the Asia Cohort Consortium (ACC) created the Reproductive Factor Working Group (WG) to offer a shared resource for investigators working with ACC data. The primary objective of this WG is to promote a common understanding of reproductive variables and their appropriate use in data analyses, which can help to ensure consistency and accuracy in research, and ultimately accelerate the pace of scientific progress.

Overall, the Reproductive Factor WG plays a critical role in ensuring that the reproductive variables collected from different cohorts within the ACC are harmonized and cleaned in a consistent and rigorous manner. This helps to generate high-quality epidemiological data that can support research to provide important insights into the health of populations in Asia and beyond.

There is a specific process for harmonizing reproductive factor variables across studies. The group ensures that data collected from different cohorts is consistent, comparable, accurate and can be combined and analysed in a consistent and meaningful way. Here are the key steps involved in harmonizing reproductive factor variables within the ACC:

- **At the beginning**: After approval by the ACC executive committee, the aims of the proposed Reproductive Factor WG were circulated. Subsequent zoom meetings, E-mails, intensive collaboration, and communication among all involved members led to the development of a Reproductive Factor WG protocol, specifying the analysis plan, and requested variables.
- **Identification of core variables**: Along with the WG leaders, Coordinating Center (CC) staff identified a set of core variables related to reproductive factors that are common and/or available across all studies. A data request template was sent to participating cohorts, soliciting information on reproductive and hormonal variables for each cohort (baseline questionnaire data only). The core variables identified were:
    - Parity status
    - Number of deliveries/pregnancies
    - Age at first delivery/pregnancy
    - Age at menarche
    - Menopausal status & Age at menopause
    - Breastfeeding
    - Use of oral contraceptives (OC)
    - Use of menopausal hormones
    - Hysterectomy

At the timepoint when data harmonization was planned, there were 13 cohorts who had reproductive factor variables available:

- SWHS: Shanghai Women's Health Study
- JACC: Japan Collaborative Cohort Study
- JPHC1: Japan Public Health Center-based prospective Study 1
- JPHC2: Japan Public Health Center-based prospective Study 2
- Miyagi: Miyagi Cohort
- Ohsaki: Ohsaki National Health Insurance Cohort Study
- LSS: Life Span Study Cohort

© 2023 Katagiri R et al. *JAMA Network Open.*

− Takayama: Takayama Study
− 3 Pref Miyagi: 3-Prefecture Miyagi Cohort
− KMCC: Korean Multi-center Cancer Cohort Study
− KNCC: Korean National Cancer Centre Cohort
− Namwon: The Namwon Study
− SCHS: Singapore Chinese Health Study

• **Standardization of variable definitions**: Comparability of reproductive variables from participating cohorts was assessed and how to devise a standardized definition for each reproductive variable was examined. Details are as follows:

− *Parity status*

Parity status at baseline was available for all cohorts except for LSS .

− *Number of deliveries/pregnancies*

Except for the LSS cohort, data on number of deliveries/pregnancies were available for all cohorts. Not all cohorts had a single variable to describe number of deliveries/pregnancies information. Many cohorts had more than one variable for the same data. Except for the SCHS cohort, all the cohorts provided number of deliveries/pregnancies information as continuous values.

− Age at menarche

All cohorts had age at menarche data as continuous values except for Takayama and SCHS.

− *Menopausal status & age at menopause*

There was a modest amount of missing data for menopausal status, particularly in LSS, JACC and KNCC studies. Implausible values for menopausal status were judged by comparing it with a participant's baseline age and/or their age at menopause (if available), such as premenopausal women reporting an age at menopause or reporting very high baseline age such as >55 years. Implausible values for age at menopause were considered <20 years. Age at menopause was available as continuous values for all cohorts except SCHS and Takayama.

−*Age at first delivery/pregnancy*

For age at first delivery/pregnancy, all cohorts except Takayama had continuous values. JACC and LSS only had data on age at first pregnancy and this value may refer to a pregnancy that is not full-term. Plausible values for age at first delivery/pregnancy were considered in the range of 10-49 years (values outside of this range were set as missing), 49 was selected as the upper cut-off because it is the median menopausal age for both Japan and Korea cohorts.

− *Breastfeeding*

Only 7 cohorts had data on breastfeeding status (JPHC1, JPHC2, Miyagi, Ohsaki, KMCC, KNCC, Namwon); three of these cohorts also had data on breastfeeding duration (KMCC, KNCC, Namwon) at baseline. Breastfeeding duration was the cumulative duration of breastfeeding (sum for all children in months). Implausible values for breastfeeding duration would be considered as those inconsistent with the number of deliveries data.

− Use of oral contraceptives

Only a few cohorts had oral contraceptive (OC) use status (Ohsaki, KMCC, KNCC, Namwon, SCHS; OC

© 2023 Katagiri R et al. *JAMA Network Open.*

use status was computed for Miyagi using data from the OC use duration variable) and/or OC use duration (Miyagi, Ohsaki, KNCC, Namwon) reported at the study baseline. Data for OC start age were available for KNCC and Namwon and OC stop age for only Namwon. Implausible values were noted after comparing with baseline age.

− *Use of menopausal hormones*

Data on hormone replacement therapy (HRT) use were not available for JACC, JPHC 1, JPHC 2, LSS and 3 Pref Miyagi cohorts. Different variables were available with different categorizations for baseline HRT. In the Takayama cohort, data on both HRT current use and HRT ever use were available.

− *Hysterectomy*

Hysterectomy at baseline information was available for Takayama, KMCC and KNCC cohorts from two variables with overlapping data.

• **Data cleaning, processing, and harmonization of variable coding**: The coding for each reproductive variable as well as other core variables was developed by the coordinating center. This includes providing instructions on the original variables that were collected in the ACC database (baseline questionnaire data) followed by any re-coding or data processing that was necessary to create the derived variables, and any relevant exclusions or exceptions. This allows data from different cohorts to be combined and analyzed in a consistent way. Participating studies are asked to recode their own data to fit the data request template to minimize the potential for error in the recoding or understanding of variables in their original form. The WG performed a thorough data cleaning and processing to identify and correct any errors or inconsistencies. This includes checking for missing data, outliers, and logical inconsistencies in the data.

As a consensus of the WG, the common exclusion criteria for reproductive factor focused data analysis included males, missing data on gender, missing age at baseline and missing information on pregnancy and number of deliveries at baseline. Details for data cleaning, processing and harmonization of reproductive variables are as follows:

− *Parity status*

Derived variables were created for parity status (nulliparous, parous, missing). When parity status at baseline was missing, it was assigned using processed data on number of deliveries/pregnancies. Women who reported stillbirth/miscarriages were also set as parous (when this information was available). The LSS study only had data on age at first pregnancy, therefore women in this study were assigned as parous if they had reported their age at first pregnancy.

− *Number of deliveries/pregnancies*

Variables that described full term pregnancies were preferentially used when available. A derived variable was created for number of children/deliveries with categorical values (no child, 1-2, 3-4, 5+ children, missing) which included nulliparous women . A separate derived variable for number of children/deliveries was created that excluded nulliparous women.

− *Age at first delivery/pregnancy*

The derived variable for age at first delivery/pregnancy was created to have categorical values (≤ 20, 21-25, 26-30, 31+ years, missing) and was restricted to parous women. Therefore, missing included nulliparous women as well as parous women with missing age at first delivery/pregnancy. If women were missing parity

© 2023 Katagiri R et al. *JAMA Network Open.*

status, they were also assigned missing age at first delivery/pregnancy. This is to maintain consistency across the derived variables, and we also believe that information on parity status should be more reliable than information on age at first pregnancy. A second derived variable for age at first delivery/pregnancy was also created that had continuous values.

− *Age at menarche*

Derived variable for age at menarche were created with categorical values (<13, 13-14, 15-16, 17+ years, missing). Plausible values for age at menarche were considered in the range of 10-23 years (values outside of this range were set as missing) as a general consensus of the WG. A variable for age at menarche with continuous values was also created but it was missing for Takayama and SCHS cohorts who had only categorical values.

− *Menopausal status & age at menopause*

Menopausal status was assigned (when missing) using data on age at menopause (when available). When age at menopause was missing or implausible, menopausal status was assigned using data on baseline age. Considering the median menopausal age for both Japan and Korea is 49, the following baseline age cut-offs were used to assign menopausal status: Age 54+ years: postmenopausal, Age 44 years or less: premenopausal, Ages 45-53 years: perimenopausal/unknown. A derived variable for age at menopause (<45, 45-49, 50-54, 55+, missing) was created with categorical values. Premenopausal women, those missing menopausal status or those with implausible age of menopause < 20 years were set as missing age at menopause. Another derived variable for age at menopause was created with continuous values and medians of categories were assigned for SCHS (only categorical age at menopause data was available in SCHS).

− *Breastfeeding*

New variables for breastfeeding status (never, ever, missing) and breastfeeding duration were computed. The derived breastfeeding duration variable was measured in months and had continuous values. Missing values on breastfeeding status and duration were assigned to participants who were missing information on breastfeeding status and/or breastfeeding duration (when relevant), nulliparous women and participants who were missing parity. Implausibly high values for breastfeeding duration for a small number of KMCC participants were set as missing after identifying inconsistencies after cross checking with number of deliveries.

− *Use of oral contraceptives (OCs)*

OC start/stop age variables were not cleaned because very few cohorts had those data. New variables were derived for OC ever use status (never, ever, missing) and for OC ever use duration (as continuous values). For OC ever use status, missing was assigned to missing OC use status. Conflicting information of OC use and duration, such as never users reporting duration or ever users reporting no duration or implausible values after cross checking with baseline age were assigned as missing.

− *Use of postmenopausal hormones (Hormone replacement therapy or HRT)*

New variables were derived for HRT ever use status (never, ever, missing) and postmenopausal HRT ever use status (restricted to postmenopausal women). Those participants with missing values were those with missing information on HRT ever use status.

− *Hysterectomy*

© 2023 Katagiri R et al. *JAMA Network Open.*

A derived variable for hysterectomy status was created (never, ever, missing). Missing was set for participants with missing/unknown information on hysterectomy status and if data gave a conflicting answer.

- **Quality control**: The WG has a robust system of quality control in place to ensure that the data remains accurate and up to date. This includes regular audits, data validation, and monitoring of data quality over time.

© 2023 Katagiri R et al. *JAMA Network Open.*
