# Supplementary material for: Reproductive Factors and Endometrial Cancer Risk Among Women
Source: JAMA Netw Open. 2023 Sep 5;6(9):e2332296. doi: 10.1001/jamanetworkopen.2023.32296 (PMC10481237; doi:10.1001/jamanetworkopen.2023.32296)
Supplement: Supplement 2. — Data Sharing Statement [file jamanetwopen-e2332296-s002.pdf]

Data Sharing Statement

Katagiri. Reproductive Factors and Endometrial Cancer Risk Among Women. *JAMA Netw Open*. Published September 05, 2023. doi:10.1001/jamanetworkopen.2023.32296

Data
Data available: No

Additional Information
Explanation for why data not available: The data can be made available upon reasonable request.
